# Quality of Reporting Randomized Controlled Trials Published in Three of the Most Citable Periodontal Journals from 2018 to 2022

**DOI:** 10.3390/healthcare11243180

**Published:** 2023-12-16

**Authors:** Fahad Alharbi, Khalid Gufran, Muzammil Moin Ahmed, Abdulaziz Alsakr, Abdullah Almutairi

**Affiliations:** 1 Department of Preventive Dental Sciences, College of Dentistry, Prince Sattam bin Abdulaziz University, Al-Kharj 11942, Saudi Arabia; fahad.alharbi@psau.edu.sa (F.A.); a.alsakr@psau.edu.sa (A.A.); 2 Department of Dental and Oral Health, College of Applied Health Sciences, Qassim University, Al Rass 51921, Saudi Arabia; mu.ahmed@qu.edu.sa; 3 Department of Periodontology and Oral Medicine, College of Dentistry, Qassim University, Buraydah 52571, Saudi Arabia; dr.almutairi@qu.edu.sa

**Keywords:** CONSORT, periodontology, quality reporting, RCTs

## Abstract

This study aimed to evaluate the reporting quality of randomized clinical trials (RCTs) in periodontology. Three leading periodontology journals, the Journal of Periodontology (JOP), the Journal of Clinical Periodontology (JOCP), and the Journal of Periodontal Research (JOPR), were selected for this investigation. The RCTs were identified by manually searching for human trial articles published in these three journals. Two authors independently conducted the literature search, and a pre-piloted extraction sheet was used to screen the potential RCTs. The CONSORT checklist guidelines were employed to calculate the score value. Intra-examiner reliability was assessed by scoring a random sample of 10% of the papers in a second round conducted by the first examiner three months after the initial data collection. A search of abstracts published over a five-year period yielded 176 articles that reported RCTs, accounting for 11.7% of all articles published in the three journals. The highest number of RCTs was published in 2020, and more than half of the included RCTs (51%) originated from Europe. Many of the analyzed RCTs inadequately reported almost half of the items on the CONSORT checklist. Furthermore, univariate analysis revealed significant associations between certain factors and the overall CONSORT score, such as publication in JOP (*p* = 0.048), publication year of 2019 (*p* = 0.041) and 2021 (*p* = 0.042), first author from North America (*p* = 0.016), and RCTs with more than six authors (*p* = 0.042). Clinical trial research in periodontics has made significant progress in the past five years. However, there is room for improvement in adhering to the CONSORT guidelines.

## 1. Introduction

Evaluating treatments and drawing reliable conclusions regarding suggested treatment modalities are important goals of research studies in the field of dentistry. For therapeutic, diagnostic, and prognostic problems, there are hierarchies of evidence, and the randomized controlled trial is at the top of the list [1]. The best type of evidence in medical research is provided through randomized controlled trials (RCTs) [2].

The RCTs are highly regarded as the strongest form of experimental support for clinical practice. They provide a solid foundation for reliable systematic reviews and meta-analyses, which are considered the most robust types of evidence to guide optimal therapeutic care. The key feature of the RCTs is randomization, which, when properly implemented, significantly minimizes bias, and prevents biases from other sources like allocation, attrition, performance, and assessment. Additionally, the RCTs are effective in demonstrating cause-and-effect relationships, adding to their significance in the field of research [3]. Careful planning and execution are crucial for conducting high-quality RCTs that provide accurate and precise clinical results. The reliability and usefulness of RCTs depend on how well they are conducted, including the methods used, the study design, and the interpretation of the findings. To ensure that RCTs are reliable, it is important to report every part of the study accurately and thoroughly [4]. In recognition of this importance, distinguished journal editors, reviewers, and statisticians have endeavored to uphold the highest reporting standards for RCTs. As a result of their efforts, the Consolidated Standards of Reporting Trials (CONSORT) guidelines were developed [3,5]. These guidelines aim to promote comprehensive and transparent reporting of RCTs, elevating the overall quality and integrity of research publications.

The CONSORT guidelines were initially introduced in 1996 and subsequently updated in 2001 and 2010 [3,6]. They consist of a flowchart and a set of components that must be included when reporting the results of an RCT. However, achieving good reporting has proven to be a challenge across different dental fields. Studies conducted after the initial release of the CONSORT statement revealed that the standard of RCT reporting fell below the required level [7]. Various dental specialties, including periodontology, prosthodontics, implantology, pedodontics, orthodontics, and public health dentistry, have conducted assessments to evaluate the quality of RCTs [8].

In the field of periodontology, there has been a growing interest in evaluating the reporting quality of randomized clinical trials (RCTs) through several recent studies. These studies have shown that although there has been some improvement over the years, the reporting quality of periodontology RCTs still falls short of the optimal standard [6,9]. Given that RCTs are considered the gold standard of evidence, it is crucial to assess their quality in the field of periodontology. However, there have been very few studies conducted in the past to evaluate the quality of RCTs in the field of periodontics. Therefore, the objective of the current study was to assess the reporting quality of clinical trials in periodontology.

## 2. Materials and Methods

Three of the most citable periodontology journals were chosen for this study: the Journal of Periodontology (JOP), the Journal of Clinical Periodontology (JOCP), and the Journal of Periodontal Research (JOPR). These journals were selected based on the assumption that they have stricter criteria for publishing reports of randomized controlled trials (RCTs) compared to other journals in the field. The RCTs were identified through a hand search of all human trial articles published between January 2018 and June 2022 in these three journals. This study excluded in vitro studies, laboratory-based trials, and conference abstracts. The keywords “randomized controlled trial”, “randomized controlled trial”, “assigned”, and “prospective or “comparative” appeared in the titles and abstracts of the eligible RCTs, or it was clear from the methodology that the study was a randomized clinical trial. After that, the full texts of all articles that met the inclusion criteria were obtained. Two authors (AA and FA) conducted the literature search independently and in duplicate; any disagreement was resolved through an open discussion between the authors until a mutual agreement was reached. Using a pre-piloted extraction sheet, one author (AA) screened the potential RCTs. The CONSORT checklist guidelines were used to calculate the score value [10]. For each item, a scoring system was employed, where ‘Yes’ indicated applicability and was assigned a score of “1”, ‘No’ denoted absence and was assigned a score of “0”, and ‘NA’ indicated inapplicability and not included in the final score calculation [11,12]. In cases where the research question of a study made certain items inapplicable, such as blinding patients or treating clinicians in the RCTs assessing intervention efficacy, they were labeled as ‘Not Applicable.’ The total score for each trial was subsequently calculated and converted to a percentage using the following equation:

Total score = (total number of ‘Yes’ items/[37-total number of ‘NA’ items])/100.

In addition to the primary data, supplementary information such as the number of authors, the continent and country of the first author, and the clinical setting of the trial were collected for each article. To ensure consistency, the authors underwent calibration by jointly scoring 10% of the included articles using the CONSORT checklist and referring to the associated explanations. A second examiner (FA) scored a random sample of 10% of the papers to assess the inter-examiner reliability of the CONSORT scores. To assess intra-examiner reliability, another random sample of 10% of the papers was scored in a second round by the first examiner (AA) three months after the initial data collection was completed.

### Statistical Analysis

Descriptive statistics and the percentage of compliance with CONSORT checklist items were reported for the included RCTs. Univariate linear regression analysis was conducted using SPSS 22.00 (IBM Co., Armonk, NY, USA), version 29, to identify variables associated with the mean CONSORT score. Inter and intra-examiner reliability was assessed with the inter-correlation coefficient (ICC) tests.

## 3. Results

The scoring of the included articles’ reporting demonstrated high levels of inter- and intra-reliability, with ICC test results indicating values of 0.95 and 0.88, respectively.

Out of 1596 total articles, the search of all abstracts of publications published over the course of five years yielded 176 articles reporting RCTs representing 11.7% of all articles published in the three journals. A total of 12 papers (7%), 67 papers (38%), and 97 papers (55%) were contributed by the JOPR, the JOP, and the JOCP, respectively. The greatest number of RCTs was published in the year 2020 and the least number of RCTs was published in 2022. The majority of the RCTs consist of four to six authors (49%) and 96% of authors work in academia. Similarly, the majority of the included studies (87%) were conducted in university settings, with only 5% conducted in private clinic settings. However, a statistician’s explicit involvement in the research team was only present in a very small number of RCTs (19%). In terms of the continent of origin of the first author, more than half of the included RCTs (51%) were published in Europe and only one RCT was published in the African region. Table 1 summarizes the general characteristics of the included randomized clinical trials.

The overall mean CONSORT score for all included RCTs was 67.7% (95% CI: 66.3 to 69.1), with the 12 RCTs published in the JOPR achieving the highest score (72.3; 95% CI: 68.7 to 75.8). Many of the RCTs included in this analysis did not adequately report nearly half of the items on the CONSORT checklist with reference to the items on the checklist. For instance, only 68.3% of the reports included information methods to generate random allocation, blinding (which was only reported in 50.3% of all reports), similarity of the intervention (0.5% of reports), harms (14.1% of reports), trial limitations (42.5% of reports), and protocols (0.9%). However, the remainder of the CONSORT checklist items were generally adequately documented in the trials (70.4–100%) (Table 2).

According to the univariate analysis, RCTs that are published in JOP (*p* = 0.048), in the years 2019 (*p* = 0.041) and 2021 (*p* = 0.042), had a first author belonged to the North American continent (*p* = 0.016) and RCTs with less than six authors (*p* = 0.042) were significantly associated with the greater overall CONSORT score (Table 3).

## 4. Discussion

Previous literature has extensively discussed the consequences of inadequate reporting in medical research and the importance of adhering to reporting guidelines [13]. Various studies have evaluated the reporting quality across different fields of dentistry [14,15,16]. To evaluate the reporting quality of recently published randomized controlled trials (RCTs), the study selected three of the most citable periodontology journals. These journals were analyzed using the CONSORT guideline. It is important to note that the impact factor (IF) of a journal does not directly reflect the quality of its published research. However, the IF is widely accepted and regarded as a benchmark, despite its limitations. It offers a measurable evaluation of a journal’s relative strength, considering factors such as peer opinions and citation rates.

Despite the establishment of the CONSORT checklist to ensure proper reporting of clinical trials, a considerable number of trials published in reputable journals still fall short of adequate reporting [12]. Furthermore, most previous studies have consistently concluded that the quality of published trials does not meet the highest standards and have recommended strict adherence to the CONSORT guideline [17,18,19].

The demographics of the 176 published RCTs in three different periodontal journals were assessed in the current study, revealing that the majority of the RCTs were published in the JOCP journal over the past five years. This finding aligns with a previous study conducted from 2015 to 2018 [20]. When comparing the number of RCTs to previous studies [20], it becomes evident that the JOPR journal had the fewest number of RCTs published since 2015, in contrast to JOP and JOCP. Interestingly, a significant increase in the number of RCTs was observed in 2020, with a similar trend observed in 2018 and 2021. It was surprising to note that despite the limitations imposed by COVID-19, a substantial number of RCTs were published in 2020 and 2021. RCTs with four to six authors were found to be more prevalent, which is consistent with the findings of previous studies [15,20]. Furthermore, it was observed that the majority of RCTs were conducted in the European continent, which aligns with the findings of the previous study [20]. However, Papageorgiou et al. [15] reported that most RCTs were conducted in Asia. It is worth noting that Papageorgiou et al. [15] assessed the quality of RCTs in 2017 and 2018, which justifies the discrepancies observed between their study and the current investigation.

The title and abstract of a scientific paper play a crucial role in conveying the essence of the entire manuscript. It is, therefore, essential to construct a title and abstract that accurately reflects the content of the study. The CONSORT checklist emphasizes the inclusion of RCT identification in the title. This is important because, during electronic database searches for research purposes, the absence of the study type mentioned in the title may unintentionally exclude relevant RCTs [20]. In the present study, all the studies included in the three journals had RCT mentioned in their titles. A similar finding was also observed in a previous study that assessed the quality of RCTs using the same three journals [20].

To achieve sufficient study power, it is essential to perform accurate sample size calculations. Proper calculation of the study sample size enhances the credibility of the research, as it helps avoid type II errors that may lead to the rejection of alternative hypotheses [21]. It is important to differentiate between the proper reporting of a sample size calculation and merely discussing the calculation. A study reported that the RCTs published in high-impact medical journals inadequately conducted sample size calculations and often reported calculations based on assumptions, which is incorrect [22]. Similarly, a lack of power analysis has been observed in periodontal and implant journals as well [23,24]. Furthermore, Jokstad et al. [25] examined 92 RCTs in the prosthodontics journal and found that only nine of them properly conducted sample size calculations. Similarly, when six major clinical dental specialty journals were assessed, only 7.3% were found to have proper sample size calculations [26]. In comparison, the current study demonstrated comparatively better results in terms of sample size calculations, with 76.5% of studies from all three journals conducting appropriate sample size calculations.

The randomization process is crucial in research to minimize confounding and selection bias [27]. However, previous literature has identified a lack of proper reporting and inadequate details regarding randomization procedures [28,29,30,31]. In the CONSORT checklist, items 8a and 8b (Table 2) specifically address the randomization method and types of randomizations. Unfortunately, in the current study, only 68.3% and 59.5% of the RCTs from all three journals reported these items properly. This finding is consistent with a previous study that evaluated the RCTs from 2015 to 2018, where only 8% of the RCTs from the same three journals reported appropriate randomization processes [20]. Montenegro et al. [32] also found a lower percentage of reporting on randomization in periodontal journals. Furthermore, a previous study indicated that less than one-third of RCTs published in various fields of dentistry adequately reported the randomization process [28]. These findings highlight the conflicting outcomes of previous RCTs, underscoring the importance of adhering to the randomization and allocation processes as integral components of the CONSORT checklist.

The blinding technique is highly advantageous in clinical trials, as it helps ensure the most reliable and unbiased results, especially when evaluating subjective outcomes. Lack of blinding could lead to inflated treatment effects [33]. However, it is important to acknowledge that achieving blinding of clinicians or patients in periodontic treatments can present practical challenges. In such situations, a viable solution is to involve an independent third party who carries out measurements without any knowledge of the treatment protocol or patient group distribution [15]. In the present study, only 50.3% of the RCTs reported implementing blinding procedures. Previous studies reported lower percentages of clinician blinding (9%), patient blinding (8%), and assessment blinding (10%) compared to the current study [20]. These findings highlight the need to improve the implementation of blinding techniques in periodontal trials to enhance the reliability and validity of the study outcomes.

Another crucial aspect of the CONSORT checklist is the registration of the RCTs. Registering a trial in the public domain enhances trial accountability and reflects the transparency of the methods employed in the clinical trial [34]. Early registration, before commencing the trial, helps mitigate biases associated with non-publication, delayed publication, or duplicate publication [35]. However, it has been observed that many RCTs are registered retrospectively, either after the trial has commenced or before publication when journals require a registration number [15]. Previous studies have indicated that a significant proportion of published RCTs in periodontal journals were not registered in any public domain [15,20]. Nevertheless, the current study demonstrates a noteworthy improvement, with 81.9% of the identified trials across all three journals being registered. This finding suggests that authors have increasingly embraced the CONSORT guidelines in recent years, recognizing the importance of adhering to proper RCT practices.

One limitation of this study is its narrow focus on the reporting quality of clinical trials within the field of periodontology and its reliance on three specific periodontology journals. While these journals are recognized for their credibility and contribution to the field of periodontology, they may not fully represent the reporting quality in other dental or medical specialties. Different specialties/journals may have unique considerations and reporting practices that were not accounted for in this study. Therefore, the findings should be interpreted with caution when attempting to generalize them to other areas of healthcare.

Moreover, the study’s evaluation was limited to randomized clinical trials published within a five-year timeframe. While a five-year period provides insight into recent reporting practices, it may not capture the complete spectrum of reporting quality over a longer duration. Reporting practices may have evolved or improved before the selected timeframe or may continue to evolve beyond it. Therefore, the findings may not fully reflect the current state or trends in reporting the quality of clinical trials in periodontology.

Additionally, although the study identified associations between certain factors (such as publication in specific journals, publication year, author affiliation, and number of authors) and the overall CONSORT score, it is important to note that these associations do not imply causation. Other unmeasured factors, such as the expertise of the research teams, funding sources, or institutional guidelines, may have influenced the reporting quality of the included RCTs. Further research is needed to investigate these potential factors and their impact on reporting quality in periodontology and other fields of study.

## 5. Conclusions

Clinical trial research in periodontics has made significant advancements in the last five years. However, there is still room for improvement in adhering to the CONSORT guideline. It is crucial to maximize the benefits derived from clinical trials by fostering collaborative efforts among journal editors, peer reviewers, and authors to ensure the publication of comprehensive trial reports.

## Figures and Tables

**Table 1:** General characteristics of the included randomized clinical trials.

Characteristic	Number of Publications	%	Mean Score	SD	95% CI	
					Lower	Upper
**Journals**						
JOP	67	38%	65.6	9.2	63.3	67.8
JOCP	97	55%	68.58	9.643	66.6	70.5
JOPR	12	7%	72.3	5.6	68.7	75.8
**Year**						
2018	18	10%	73.5	7.1	70.0	77.0
2019	42	24%	65.4	8.8	62.31	68.6
2020	51	29%	69.4	8.54	66.9	71.9
2021	48	27%	65.6	9.1	62.9	68.2
2022	17	10%	68.0	10.2	62.8	73.3
**Authors**						
>4	21	12%	66.49	10.25	61.8	71.2
4 to 6	87	49%	66.51	10.18	64.3	68.7
<6	68	39%	69.9	7.9	67.7	71.5
**Settings**						
Private	9	5%	66.0	8.0	59.8	72.1
University	153	87%	67.7	9.4	66.2	69.2
Governmental	1	1%	75.7	-	-	-
Mixed	13	7%	68.8	10.9	62.2	75.4
**Work in Academia**						
No	7	4%	63.2	6.6	57.1	69.3
Yes	169	96%	67.9	9.5	66.5	69.3
**Statistician Involvement**						
No	142	81%	68.4	8.8	66.9	69.8
Yes	34	19%	64.9	11.5	60.9	68.9
**Continent**						
Asia	33	19%	69.21	10.045	65.7	72.8
Africa	1	1%	64.86	-	-	-
North America	27	15%	63.1	7.421	60.2	66.0
South America	25	14%	69.49	9.894	65.4	73.6
Europe	90	51%	68.08	9.378	66.1	70.0
Overall	176	100%	67.7	9.4	66.3	69.1

JOP; the Journal of Periodontology, JOP; Journal of Periodontology, JOCP; Journal of Clinical Periodontics, JOPR; Journal of Periodontal Research, SD; standard deviation, %; percentage, CI; confidence interval.

**Table 2:** Calculated score value of the CONSORT checklist guideline.

Section	Item No.	Checklist	All Journals	JOP	JOCP	JORP
Title and abstract	1a	Identification as a randomized trial in the title	100%	100.0%	100.0%	100.0%
	1b	Structured summary of trial design, methods, results, and conclusions (for specific guidance see CONSORT for abstracts)	97.2%	95.5%	99.0%	100.0%
Introduction Background and objectives	2a	Scientific background and explanation of the rationale	98.9%	100.0%	100.0%	100.0%
	2b	Specific objectives or hypotheses	97.8%	98.5%	100.0%	100.0%
Methods Trial design	3a	Description of trial design (such as parallel, factorial) including allocation ratio	88.3%	83.6%	93.8%	100.0%
	3b	Important changes to methods after trial commencement (such as eligibility criteria), with reasons	4.4%	10.4%	1.0%	0.0%
Participations	4a	Eligibility criteria for participants	96.7%	100.0%	100.0%	100.0%
	4b	Settings and locations where the data were collected	95.6%	98.5%	100.0%	100.0%
Interventions	5	The interventions for each group with sufficient details to allow replication, including how and when they were administered	95.7%	100.0%	100.0%	100.0%
Outcomes	6a	Completely defined pre-specified primary and secondary outcome measures, including how and when they were assessed	94.6%	98.5%	100.0%	100.0%
	6b	Any changes to trial outcomes after the trial commenced, with reasons	0.0%	0.0%	0.0%	0.0%
Sample size	7a	How sample size was determined	76.5%	77.6%	82.5%	91.7%
	7b	When applicable, explanation of any interim analyses and stopping guidelines	4.8%	10.4%	1.0%	8.3%
Randomization Sequence generation	8a	The method used to generate the random allocation sequence	68.3%	65.7%	75.3%	100.0%
	8b	Type of randomization; details of any restriction (such as blocking and block size)	59.5%	50.7%	71.1%	83.3%
Allocation concealment mechanism	9	The mechanism used to implement the random allocation sequence (such as sequentially numbered containers), describing any steps taken to conceal the sequence until interventions were assigned	57.6%	55.2%	69.1%	50.0%
Implementation	10	Who generated the random allocation sequence, who enrolled participants, and who assigned participants to interventions	53.6%	52.2%	62.9%	58.3%
Blinding	11a	If done, who was blinded after assignment to interventions (for example, participants, care providers, those assessing outcomes) and how	50.3%	65.7%	49.5%	41.7%
	11b	If relevant, a description of the similarity of interventions	0.5%	0.0%	1.0%	0.0%
Statistical methods	12a	Statistical methods used to compare groups for primary and secondary outcomes	89.7%	100.0%	99.0%	100.0%
	12b	Methods for additional analyses, such as subgroup analyses and adjusted analyses	70.4%	73.1%	79.4%	100.0%
Results Participant flow (a diagram is strongly recommended)	13a	For each group, the number of participants who were randomly assigned received the intended treatment and were analyzed for the primary outcome	63.5%	62.7%	74.2%	91.7%
	13b	For each group, losses and exclusions after randomization, together with reasons	63.1%	62.7%	74.2%	91.7%
Recruitment	14a	Dates defining the periods of recruitment and follow-up	73.4%	92.5%	74.2%	100.0%
	14b	Why the trial ended or was stopped	1.5%	3.0%	1.0%	0.0%
Baseline data	15	A table showing baseline demographic and clinical characteristics for each group	52.7%	37.3%	73.2%	83.3%
Number analysed	16	For each group, the number of participants (denominator) included in each analysis and whether the analysis was by originally assigned groups	85.6%	97.0%	99.0%	100.0%
Outcomes and estimation	17a	For each primary and secondary outcome, results for each group, and the estimated effect size and its precision (such as 95% confidence interval)	85.2%	98.5%	97.9%	100.0%
	17b	For binary outcomes, presentation of both absolute and relative effect sizes is recommended	61.3%	53.7%	81.4%	83.3%
Ancillary analyses	18	Results of any other analyses performed, including subgroup analyses and adjusted analyses, distinguishing pre-specified from exploratory	37.1%	46.3%	41.2%	41.7%
Harms	19	All important harms or unintended effects in each group (for specific guidance see CONSORT for harms)	14.1%	17.9%	14.4%	25.0%
Discussion Limitations	20	Trial limitations, addressing sources of potential bias, imprecision, and, if relevant, multiplicity of analyses	42.5%	43.3%	54.6%	50.0%
Generalizability	21	Generalizability (external validity, applicability) of the trial findings	66.8%	94.0%	69.1%	75.0%
Interpretation	22	Interpretation consistent with results, balancing benefits and harms, and considering other relevant evidence	75.6%	77.6%	96.9%	100.0%
Other information Registration	23	Registration number and name of trial registry	81.9%	100.0%	95.9%	100.0%
Protocol	24	Where the full trial protocol can be accessed, if available	0.9%	3.0%	0.0%	0.0%
Funding	25	Sources of funding and other support (such as the supply of drugs), the role of funders	76.9%		95.9%	100.0%

JOP; the Journal of Periodontology, JOP; Journal of Periodontology, JOCP; Journal of Clinical Periodontics, JOPR; Journal of Periodontal Research, %; percentage.

**Table 3:** Linear regression analysis for quality evaluation, with the overall CONSORT score.

Variables	B	95% CI		*p*
		Lower	Upper	
**Journal**				
JOCP	Baseline reference			
JOP	−2.9	−5.9	−0.01	0.048 *
JORP	3.7	−1.9	9.3	0.193
**Year of publication**				
2020	Baseline reference			
2018	4.1	−0.9	9.1	0.107
2019	−3.9	−7.7	−0.2	0.041 *
2021	−3.8	−7.4	−0.1	0.042 *
2022	−1.3	−6.4	3.7	0.602
**Continents**				
Europe	Baseline reference			
Asia	1.1	−2.6	4.9	0.549
Africa	−3.2	−0.217	0.153	0.732
North America	−5	−9	−0.9	0.016 *
South America	1.4	−2.7	5.6	0.503
**List of Authors**				
Four to six	Baseline reference			
Fewer than four	0	−4.5	4.5	0.992
More than six	−3.1	0.1	6.1	0.042 *

B; coefficient, CI; confidence interval, %; percentage, *p*; *p*-value, *; significant difference (*p* < 0.05), JOP; the Journal of Periodontology, JOP; Journal of Periodontology, JOCP; Journal of Clinical Periodontics, JOPR; Journal of Periodontal Research.

## Data Availability

Data are contained within the article.
